# Severe pneumonia with pleural effusion caused by co-infection of *Paecilomyces variotii* and *Penicillium oxalicum* in a diabetic patient

**DOI:** 10.1186/s12879-024-09496-6

**Published:** 2024-06-19

**Authors:** Xiuri Wang, Xingchun Chen, Yunxiao Liang, Liuyang Hu

**Affiliations:** 1 Department of Gastroenterology, The People’s Hospital of Guangxi Zhuang Autonomous Region, Guangxi Academy of Medical Sciences, Nanning, 530016 China; 2 Department of Laboratory Medicine, The People’s Hospital of Guangxi Zhuang Autonomous Region, Guangxi Academy of Medical Sciences, Nanning, 530016 China

**Keywords:** *Paecilomyces variotii*, *Penicillium oxalicum*, Co-infection, Pneumonia, Pleural effusion

## Abstract

**Background paecilomyces:**

and *Penicillium* are considered as rare opportunistic pathogens in immunocompromised hosts, and pneumonia caused by *Paecilomyces* and *Penicillium* is rare. In this study, we present first case of severe pneumonia with pleural effusion caused by co-infection of *Paecilomyces variotii* (*P. variotii*) and *Penicillium oxalicum* (*P. oxalicum*) in a 66-year-old female with poorly controlled type 2 diabetes.

**Case presentation:**

A 56-year-old woman patient presented to hospital for nausea, poor appetite, and vomiting for one day. On the second day of admission, blood culture and renal puncture fluid culture grew multidrug-resistant *Escherichia coli* (imipenem/cilastatin sensitive), and she received combination therapy with imipenem/cilastatin (1 g, every 8 h) and vancomycin (0.5 g, every 12 h). On the fourth day, she developed symptoms of respiratory failure. Pulmonary computed tomography (CT) showed an increase in pneumonia compared to before, with minor pleural effusion on both sides. Two fungi were isolated repeatedly from BALF culture, which were confirmed as *P. variotii* and *P. oxalicum* by Internal transcribed spacer (ITS) sequencing. Her pleural effusion was completely absorbed, pneumonia symptoms have significantly improved and discharged with receiving liposomal amphotericin B treatment for four weeks.

**Conclusions:**

It is worth noting that clinicians and laboratory personnel should not simply consider *Paecilomyces* and *Penicillium* species as contaminants, especially in immunocompromised patients. Early fungal identification and antifungal drug sensitivity are crucial for clinical drug selection and patient prognosis.

**Supplementary Information:**

The online version contains supplementary material available at 10.1186/s12879-024-09496-6.

## Introduction

*P. variotii* is a common environmental mould that is widespread in composts, soils and food products [1]. *P. variotii* is an emerging causative agent of hyalohyphomycosis in the immunocompromised patient [1]. *P. variotii* is associated with various types of infections in humans, with the most common sites affected being the peritoneum and the lungs [2]. It has also been reported to cause bloodstream infections [3], central nervous system infections [4], infective endocarditis [5], endophthalmitis [6], skin and soft tissue infections [7]. These infections can be life-threatening, particularly in critically ill patients with weakened immune systems or those with indwelling medical devices [2]. *Penicillium* is one of the most common fungi in the environment and is rarely a pathogen in humans. However, it has been increasingly recognized as an opportunistic pathogen in immunocompromised hosts, causing endophthalmitis [8], pulmonary infections [9], endocarditis [10], osteomyelitis [11], skin infections [12], and disseminated infections [13]. We present first case of severe pneumonia with pleural effusion caused by co-infection of *P. variotii* and *P. oxalicum* in a 56-year-old female with poorly controlled type 2 diabetes.

## Case presentation

A 56-year-old woman patient presented to the emergency department of the People’s Hospital of Guangxi Zhuang Autonomous Region, China, due to nausea, poor appetite, and vomiting for one day. She had a history of poorly controlled type 2 diabetes for 5 years and hypertension for 10 years. Physical examination revealed fever, with 40 °C temperature peak, low blood pressure (63/39mmHg), and a palpable 6 × 4 cm mass in the lower abdomen. Laboratory results showed leukocytosis (24.48 × 10^9/L), thrombocytopenia (78 × 10^9/L), C-reactive protein of > 200.00 mg/L, Procalcitonin of > 50.00ng/ml, IL-6 of 532.00pg/ml, heparin binding protein of 124.8ng/ml and poor glycemic control (Glu 25.2 mmol/L, HbA1c 11.5%). She was diagnosed with diabetes ketoacidosis. Due to the severity of her condition, she was admitted to the ICU. Chest CT showed bilateral lungs minimal inflammation, and focal fibrotic opacities. Abdominal CT revealed right kidney enlargement and air accumulation, considering infectious lesion, not excluding emphysematous pyelonephritis; free gas is seen in the right kidney anterior compartment, considering lesion rupture. She received fluid infusion to improve circulation and active blood glucose control, combine with continuous pumping of norepinephrine 1ug/kg/min, hydrocortisone 2.5ug/kg/min and dobutamine 5ug/kg/min to maintain blood pressure. Cefoperazone sulbactam and teicoplanin were used for empirical anti-infection treatmen. Continuous renal replacement therapy and kidney puncture fistula surgery was performed at the bedside due to infection, reduced urine volume, acidosis, renal insufficiency and high lactate.

On the second day of admission, blood culture and renal puncture fluid culture grew multidrug-resistant *Escherichia coli* (imipenem/cilastatin sensitive), suggesting a bloodstream infection of urinary tract origin. Given the high risk of catheter-related infection due to multiple invasive procedures, the anti-infection plan was modified to include combination therapy with imipenem/cilastatin (1 g, every 8 h) and vancomycin (0.5 g, every 12 h), which can cover Gram-positive bacteria, along with continuous drainage of renal infection site and used Extracorporeal Membrane Oxygenation (ECMO) to provide continuous extracorporeal respiration and circulation for patient.

**Fig. 1:**
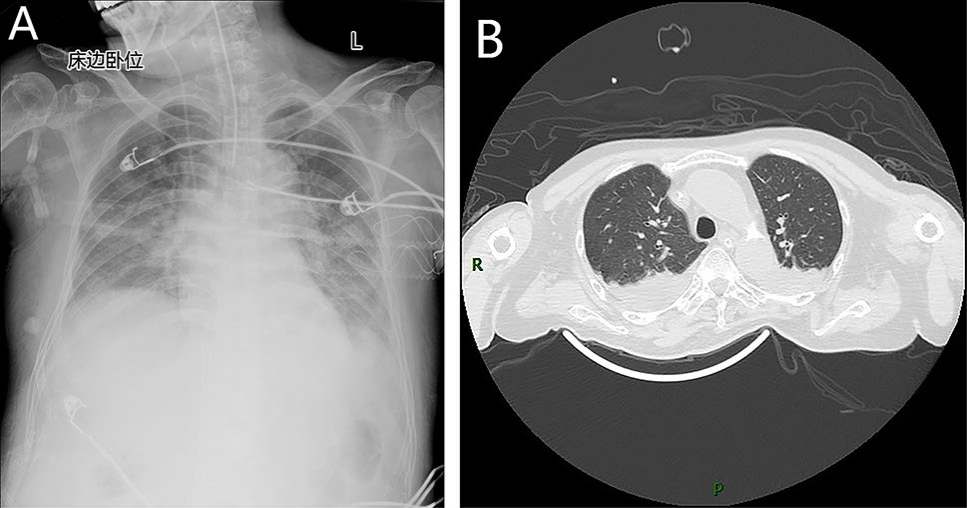
**A** Chest radiography suggested pneumonia in the right lower lung and pleural effusion on the right side. **B** Pulmonary CT showed an increase in pneumonia compared to before, with minor pleural effusion on both sides

**Fig. 2:**
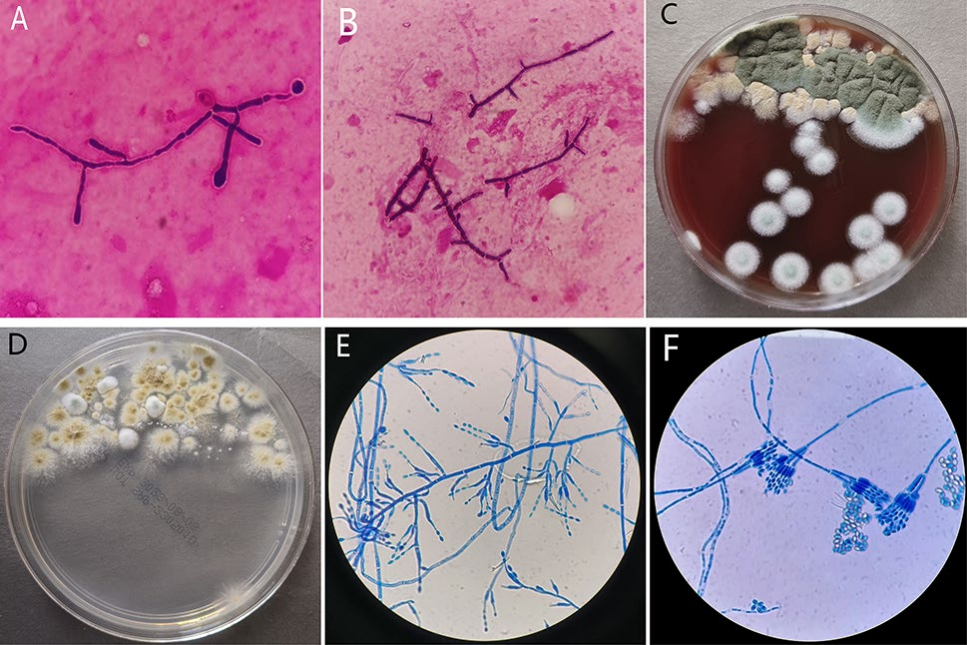
**A**, **B** The Gram staining of bronchoalveolar lavage fluid (BALF) revealed two different microscopic forms of hyphae. **C**, **D**: BALF culture showed two kinds of filamentous fungi grew on blood agar (C) and Sabouraud dextrose agar (D) after incubating at 35 °C for 48 h, of which one was yellow-brown colony, the other colony changed from white to green. **E** Lactophenol cotton blue staining of the yellow-brown colonies showed phialides are cylindrical or ellipsoidal, tapering abruptly into a long and cylindrical neck; conidia are subspherical, ellipsoidal to fusiform, hyaline, smooth-walled, and are produced in long divergent chains, which was suspected to be *P. variotii.***F** White to green colonies was suspected to be *Penicillium* based on flask-shaped phialides bear long unbranched chains of almost round conidia, with characteristic penicillus or brush appearance of the entire structure on the lactophenol cotton blue stain

On the fourth day, she developed symptoms of respiratory failure. Chest radiography suggested pneumonia in the right lower lung and pleural effusion on the right side (Fig. 1A). Pulmonary CT showed an increase in pneumonia compared to before, with minor pleural effusion on both sides (Fig. 1B). The next day, the Gram staining of bronchoalveolar lavage fluid (BALF) revealed two different microscopic forms of hyphae (Fig. 2A and B). BALF culture showed two kinds of filamentous fungi grew on blood agar (Fig. 2C) and Sabouraud dextrose agar (Fig. 2D) after incubating at 35 °C for 48 h, of which one was yellow-brown colony (R30383), the other colony changed from white to green (R30383-1). Lactophenol cotton blue staining of the yellow-brown colony showed phialides are cylindrical or ellipsoidal, tapering abruptly into a long and cylindrical neck; conidia are subspherical, ellipsoidal to fusiform, hyaline, smooth-walled, and are produced in long divergent chains, which was suspected to be *P. variotii* (Fig. 2E). White to green colonies was suspected to be *Penicillium* based on flask-shaped phialides bear long unbranched chains of almost round conidia, with characteristic penicillus or brush appearance of the entire structure on the lactophenol cotton blue stain (Fig. 2F). Voriconazole was added for antifungal treatment.

**Fig. 3:**
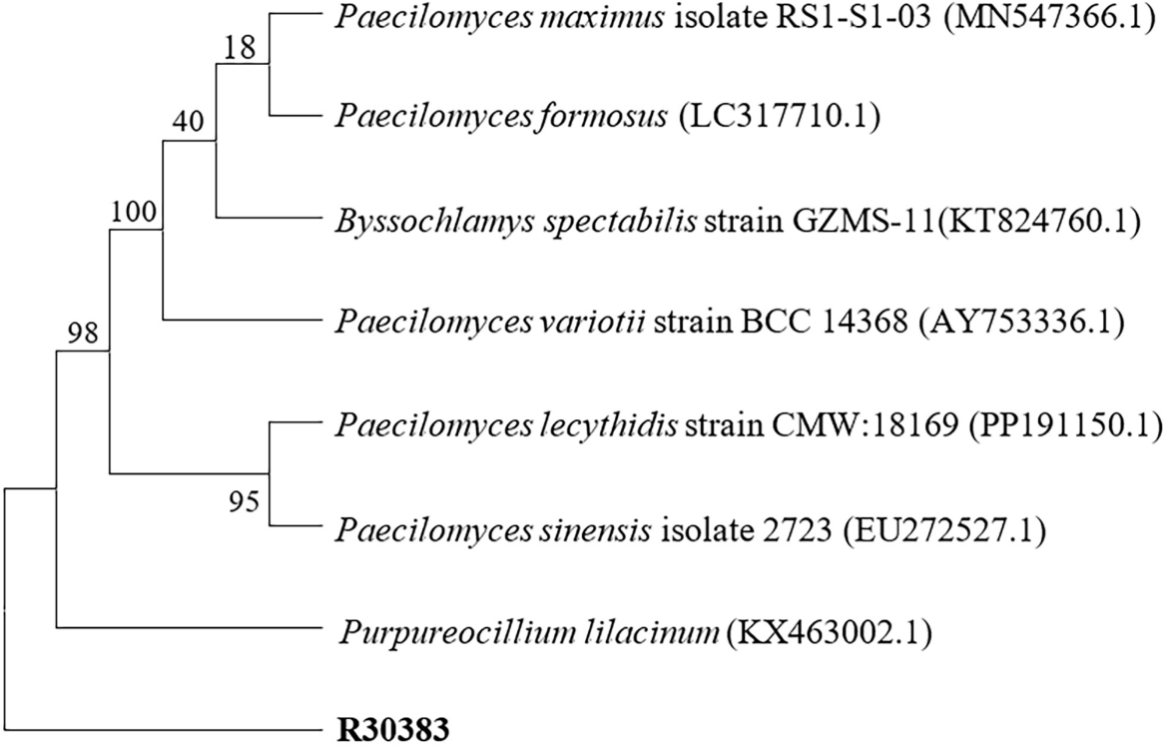
Maximum-likelihood phylogenetic tree based on the ITS sequences showing the relationship of isolated strain R30383 with members within *Paecilomyces* genus

**Fig. 4:**
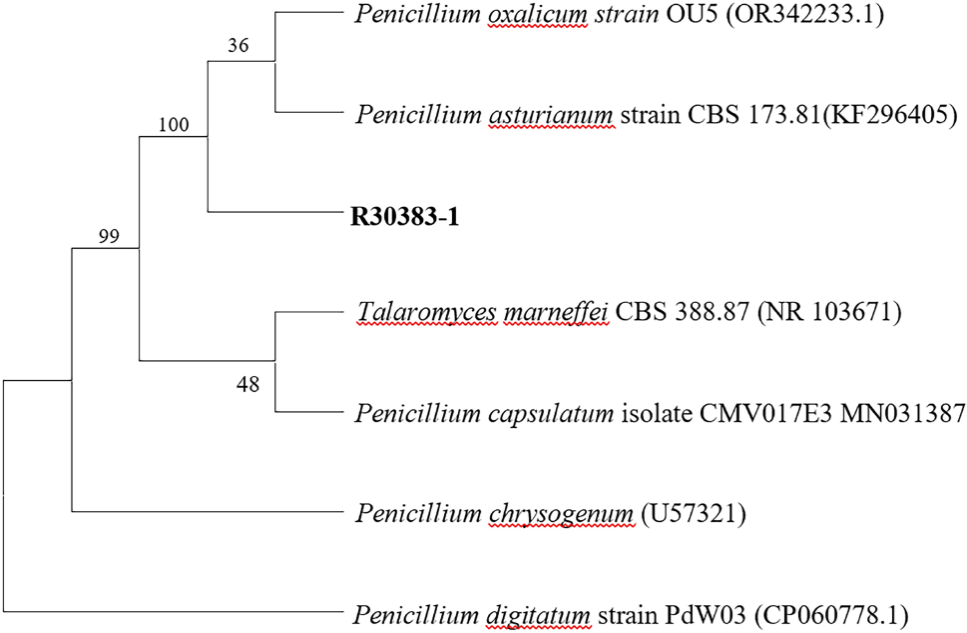
Maximum-likelihood phylogenetic tree based on the ITS sequences showing the relationship of isolated strain R30383-1 with members within *Penicillium* genus

The isolates obtained were identified by Internal transcribed spacer (ITS) sequencing. DNA extraction according to the manufacturer’s protocols of the fungal genomic DNA extraction kit produced by Jiangsu Kangwei Century Biotechnology Co., Ltd. *Paecilomyces variotii* primer pair ITS1 (5′- CATACGCTCGAGGATCGGAC-3′) and ITS4 (5′- AGCGTCATTGCTAACCCTCC-3′). *Penicillium oxalicum* primer pair ITS1 (5′-TGAAGAACGCAGCGAAATGC-3′) and ITS4 (5′-CTACAGAGCGGGTGACGAAG-3′). The obtained nucleotide sequence was compared with the nearest sequence at the National Center for Biotechnology Information GenBank database. The first homology sequence presenting the highest identity of R30383 was 100% nucleotide identity with *Paecilomyces variotii* (GenBank No.AY753336.1), and R30383-1 was 100% nucleotide identity with *Penicillium oxalicum* (GenBank No.OR342233.1). Maximum-likelihood phylogenetic tree based on the ITS sequences showing the relationship of isolated strain R30383 (Fig. 3) and R30383-1 (Fig. 4) with members within their genus, respectively.

On the 7th and 8th day of admission, *P. variotii* and *P. oxalicum* were isolated repeatedly from BALF culture. However, on the 9th day of admission, it was observed that the patient was still experiencing significant difficulty in breathing and continued to have a persistently high fever. Multiple blood culture and urine culture negative. Furthermore, it was noticed that there was a noticeable progression in the severity of the pneumonia, with the consolidation and inflammation in the lungs worsening in comparison to the initial stages of the illness. Antifungal susceptibility testing was performed according to Clinical and Laboratory Standards Institute 38 methods, determining minimum inhibitory concentrations (MICs) of *P. variotii* and *Penicillium oxalicum* after 48 h incubation were illustrated in Table 1. According to the results of antifungal susceptibility, the antimicrobial treatments plan was revised to include amphotericin B cholesterol sulfate 400 mg/d (6 mg/kg/d, patient weight: 66.7 kg) for antifungal treatment. After four weeks of treatment, bilateral pneumonia had noticeably resolved compared to before, with complete absorption of pleural effusion, indicating improvement in the condition and the patient discharged.

**Table 1:** Antifungal susceptibility of *P. variotii* and *P. oxalicum* (MIC µg/mL)

Antifungal drugs	*P*. variotii	*P*. oxalicum
Fluconazole	> 256	> 256
Amphotericin B	0.5	0.25
Voriconazole	> 32	0.38
Itraconazole	0.5	0.5

## Discussion and conclusions

*Paecilomyces* spp. is rare yet clinically significant pathogen and its infection cases have been gradually increasing in recent years. *Paecilomyces* genus includes several species, with *Purpureocillium lilacinum* (synonymy: *Paecilomyces lilacinus*) and *P. variotii* being the two most commonly associated with human diseases. The most frequent predisposing factor was presence of indwelling devices, particularly peritoneal catheters and prosthetic heart valve, and other predisposing factors included hematologic malignancies, solid organ transplantation, and diabetes [2]. *P. variotii*-related prosthetic heart valve infections and peritoneal dialysis-associated infections are most commonly seen in immunocompetent hosts, but *P. variotii* pneumonia is most frequently observed in solid organ transplant patients and diabetes patients [2]. Symptoms of *P. variotii* pneumonia are often non-specific, presenting fever, dyspnea and hypoxemia, which was difficult to distinguish from other fungal infections [2, 14]. Chest CT demonstrates nodules, ground-glass patchy consolidations and pleural effusion [15, 16].

*P. lilacinus* and *P. variotii* display similar morphological characteristics, but their susceptibility to drugs differs significantly. Thus, accurate identification of the specific species is vital for effective treatment. A multicenter study of in vitro antifungal susceptibility of 70 isolates *Paecilomyces/Purpureocillium* species isolated from clinical respiratory samples illustrated all *P. lilacinum* isolates showed growth inhibition at high MICs of amphotericin B (> 8 mg/L) and high minimal effective concentrations of echinocandins [17]. In contrast, *P. variotii* was exhibited low MICs of amphotericin B (≤ 0.5 mg/L), but their response to echinocandins was drug-dependant [17]. For azole drugs, posaconazole showed the best in vitro activity against *P. lilacinum* and *P. variotii*, with MICs ≤ 0.5 mg/L. Voriconazole and itraconazole were active in vitro against *P. lilacinum*, but showed poor in vitro activity against *P. variotii* [17]. In our case, *P. variotii* is sensitive to amphotericin B, but the mic value of voriconazole is very high. Meanwhile, the patient’s symptoms did not improve during treatment with voriconazole. Based on existing in vitro and clinical data, liposomal amphotericin B is recommended as first-line monotherapy for *Paecilomyces* infection, with posaconazole as salvage therapy [18]. Therefore, using posaconazole for empirical treatment may be a good choice when definitive species identification has not been confirmed, as in vitro data indicate that posaconazole exhibits good sensitivity against *P. lilacinus* and *P. variotii* [19]. Due to the lack of treatment guidelines, the duration of treatment must be adjusted based on clinical response. The clinical characterizations of *P. variotii* pneumonia described in the medical literature (Table 2).

**Table 2:** The clinical characterizations of *P. variotii* pneumonia described in the medical literature

Year	Age/sex	Underlying conditions	Prophylaxis	Presentation	image	Diagnostic Sample	Treatment	Outcome
1992 [20]	33/M	Diabetes Mellitus	/	Nonspecific symptoms	lung infiltrate	bronchial secretion	Failed Ketoconazole→amphotericin B	Alive
2000 [21]	9.5/F	lung transplantation	itraconazole	Persistent, unproductive cough	/	BALF	liposomal amphotericin B	Dead
2005 [18]	14/M	ALL, neutropenia	voriconazole	Fever, cough, rhinorrhoea	nodular consolidations	Disseminated, blood cultures	Liposomal amphotericin B	Alive
2013 [22]	48 /F	non- Hodgkin’s lymphoma, GVHD	cyclosporine and methotrexate	cough, dyspnea, fever	nodules and ground-glass patchy consolidations	BALF	voriconazole	Dead
2015 [16]	74/F	diabetes mellitus	/	fever	pleural effusion	pleural effusion	Itraconazole	Alive
2016 [14]	59 /F	AML, GVHD, neutropenia	acyclovir, pentamidine and fluconazole	fever	ground glass nodules	BALF	Failed voriconazole→Failed amphotericin B→improved with posaconazole	Dead
2021 [23]	47/F	T-cell-lymphoma, GVHD, chronic pancytopenia, secondary adrenal insufficiency, steroid-induced hyperglycaemia	Voriconazole	fever, shortness of breath, non-productive cough	patchy ground glass opacities, micronodules, pleural effusion	BALF	Failed micafungin→Failed liposomal amphotericin B→improved with posaconazole	Alive

Clinically isolated *penicillium*, which is commonly viewed as a “contaminant”, can actually be a pathogenic organism in individuals with weakened immune systems. It is important for both clinicians and microbiologists to recognize the potential for severe and potentially fatal *penicillium* infections. A review of 45 cases of invasive fungal infections caused by *penicillium* indicated that the majority of these cases were associated with invasive pulmonary infections [13, 24–29]. The specific *penicillium* species reported to cause pulmonary infections include *P. citrinum* [13], *P. chrysogenum* [24], *P. digitatum* [25], *P. janthinellum* [26], *P. oxalicum* [27], *P. notatum* [28], and *P. capsulatum* [29]. Factors that increase the risk of infection include systemic lupus erythematosus (SLE), diabetes, hematologic malignancies, and HIV [13, 24–29]. Interestingly, *P. oxalicum*, which is typically a pathogenic organism that affects plants, has recently been identified as a causative agent of invasive fungal disease. In 2015, Anuradha Chowdhary et al. reported three cases of invasive fungal disease caused by *P. oxalicum* in patients with acute myeloid leukemia, diabetes, and chronic obstructive pulmonary disease [27]. Furthermore, a study conducted by Lyratzopoulos et al. demonstrated that *penicillium* can cause a range of lung diseases, including disseminated parenchymal infection, lobar pneumonia, localized granuloma, fungus balls, bronchiolitis obliterans, pneumonia, and pleural effusion [30].

It is worth noting that currently reported strains of *penicillium* have shown higher MICs for voriconazole, a commonly used antifungal medication [27]. Antifungal susceptibility of 39 isolates of *Penicillium* species from clinical samples in the United States showed terbinafine and the echinocandins showed the best in vitro activity against *Penicillium* species [31]. Amphotericin B showed intermediate antifungal activity, while the azoles showed various levels of activity [31]. As a result, voriconazole should not be considered as the first-line empirical treatment option for *penicillium* infections. While there are currently no standardized treatment guidelines for *penicillium* infections, liposomal amphotericin B is the preferred treatment option [15]. The potent in vitro activity of echinocandins and terbinafine against *Penicillium* species might offer a good therapeutic alternative for the treatment of infections caused by these fungi [31]. Voriconazole, posaconazole, and itraconazole may be considered as alternative treatment options if they demonstrate susceptibility to the specific *penicillium* strains in vitro [15, 31].

According to our report, this case represents the first report of pneumonia and pleural effusion caused by a mixed infection of *P. variotii* and *P. oxalicum*, with poorly controlled type 2 diabetes and indwelling catheters as the main risk factors for infection. It is worth noting that clinicians and laboratory personnel should not simply consider *Paecilomyces* and *Penicillium* species as contaminants, especially in immunocompromised patients. Early fungal identification and antifungal drug sensitivity are crucial for clinical drug selection and patient prognosis.

### Electronic supplementary material

Below is the link to the electronic supplementary material.

Supplementary Material 1

Supplementary Material 2

## Data Availability

The sequencing data of the isolated *Paecilomyces variotii* (R30383) and *Penicillium oxalicum* (R30383-1) have been deposited into the National Center for Biotechnology Information GenBank database. GenBank accession number for *Paecilomyces variotii* (R30383) nucleotide sequences was PP892052. GenBank accession number for *Penicillium oxalicum* (R30383-1) nucleotide sequences was PP892053. The datasets generated and/or analysed during the current study are available in the National Center for Biotechnology Information GenBank database.
